# Safety of cinacalcet in children and adolescents with chronic kidney disease-mineral bone disorder: systematic review and proportional meta-analysis of case series

**DOI:** 10.1007/s11255-023-03844-2

**Published:** 2023-11-15

**Authors:** Soraya Mayumi Sasaoka Zamoner, Henrique Mochida Takase, Marcia Camegaçava Riyuzo, Jacqueline Costa Teixeira Caramori, Luis Gustavo Modelli de Andrade

**Affiliations:** 1 Botucatu School of Medicine, Pediatrics Department - Pediatric Nephrology, University São Paulo State-UNESP, District of Rubiao Junior, Botucatu, SP 18618-970 Brazil; 2 Botucatu School of Medicine, Internal Medicine Department – Nephrology, University São Paulo State-UNESP, District of Rubiao Junior, Botucatu, SP 18618-970 Brazil; 3 Clinics Hospital - Botucatu School of Medicine, District of Rubiao Junior, Botucatu, SP 18618-970 Brazil

**Keywords:** Cinacalcet, Chronic kidney disease, Mineral bone disorder

## Abstract

**Background:**

Mineral and bone disease in children with chronic kidney disease can cause abnormalities in calcium, phosphorus, parathyroid hormone, and vitamin D and when left untreated can result in impaired growth, bone deformities, fractures, and vascular calcification. Cinacalcet is a calcimimetic widely used as a therapy to reduce parathyroid hormone levels in the adult population, with hypocalcemia among its side effects. The analysis of safety in the pediatric population is questioned due to the scarcity of randomized clinical trials in this group.

**Objective:**

To assess the onset of symptomatic hypocalcemia or other adverse events (serious or non-serious) with the use of cinacalcet in children and adolescents with mineral and bone disorder in chronic kidney disease.

**Data sources and study eligibility criteria:**

The bibliographic search identified 2699 references from 1927 to August/2023 (57 LILACS, 44 Web of Science, 686 PubMed, 131 Cochrane, 1246 Scopus, 535 Embase). Four references were added from the bibliography of articles found and 12 references from the gray literature (Clinical Trials). Of the 77 studies analyzed in full, 68 were excluded because they did not meet the following criteria: population, types of studies, medication, publication types and 1 article that did not present results (gray literature).

**Participants and interventions:**

There were 149 patients aged 0–18 years old with Chronic Kidney Disease and mineral bone disorder who received cinacalcet.

**Study appraisal and synthesis methods:**

Nine eligible studies were examined for study type, size, intervention, and reported outcomes.

**Results:**

There was an incidence of 0.2% of fatal adverse events and 16% of serious adverse events (*p* < 0.01 and *I*^2^ = 69%), in addition to 10.7% of hypocalcemia, totaling 45.7% of total adverse events.

**Limitations:**

There was a bias in demographic information and clinical characteristics of patients in about 50% of the studies and the majority of the studies were case series.

**Conclusions and implications of key findings:**

If used in the pediatric population, the calcimimetic cinacalcet should be carefully monitored for serum calcium levels and attention to possible adverse events, especially in children under 50 months.

**Systematic review registration number (PROSPERO register):**

CRD42019132809.

## Background

Mineral and bone disorder (MBD) is a common complication in children with chronic kidney disease (CKD), characterized by abnormalities of calcium, phosphorus, parathyroid hormone (PTH), vitamin D, fibroblast growth factor (FGF) 23, vascular calcifications, impairment of linear growth, changes in bone histology and bone deformities [1–3]. The current guideline KDIGO 2017 for the treatment of adults with CKD-MBD includes approved drugs by the US Food and Drug Administration (FDA) [4] and European Medicines Agency (EMA) such as sterols, vitamin D analogs, phosphate binders and calcimimetics. Cinacalcet is an allosteric calcium-sensing receptor (CaSR) modulator that increases the sensitivity of CaSR, especially in the parathyroid glands, to serum calcium, resulting in the suppression of PTH secretion.

In 2017, the EMA approved cinacalcet in children over 3 years of age with CKD-MBD on dialysis who did not achieve control of hyperparathyroidism with traditional therapies. Additionally, in 2020 the European Society of Pediatric Nephrology and the ERA-EDTA Group [5] published a document with 22 positions regarding the use of cinacalcet in children on dialysis. However, the FDA [4], in a recent document of 2020, has not approved the drug in the same population. The KDIGO 2017 guidelines also do not recommend the drug in children because of the scarcity of information on the safety and efficacy of cinacalcet in this population.

The aim of study was to evaluate the onset of symptomatic hypocalcemia or other adverse events (severe and non-serious) with the use of cinacalcet in children and adolescents with CKD-MBD.

## Methods

### Search strategy and study assessment

**Fig. 1:**
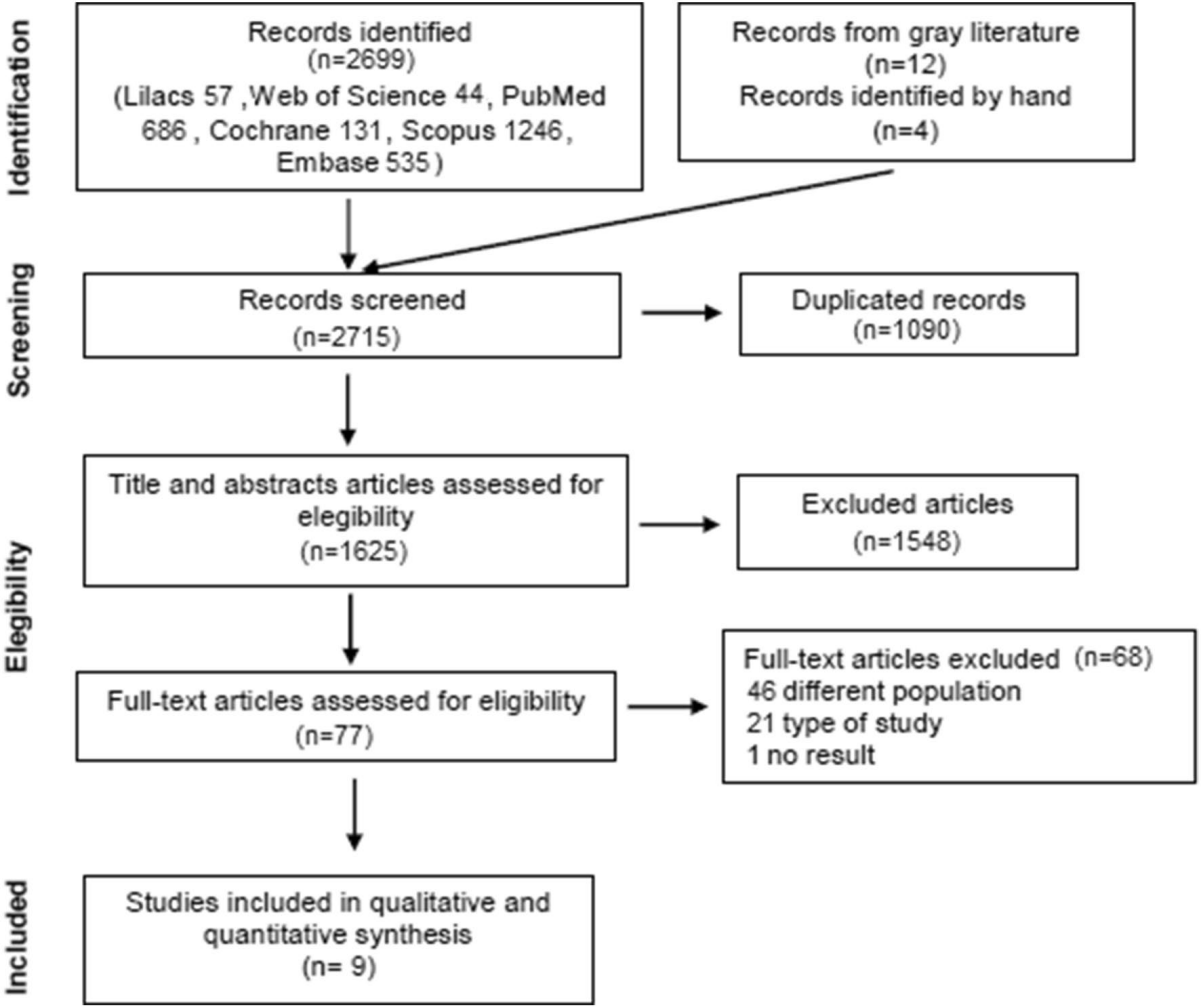
Selection of eligible papers and reasons for exclusion

A search was performed in Pubmed, Embase, Lilacs, Scopus, Web of Science and Cochrane from 1927 to August/2023 without language restriction. Keywords, “MeSH”, “Emtree terms”, DeCS and uncontrolled vocabulary were used in order to select all articles related to the use of cinacalcet. The literature search identified 2699 published articles and 16 records were added from gray literature and other references. Duplicated articles were removed, and 1548 records were excluded based on the Title or Abstract. Two independent reviewers analyzed full-text articles (*n* = 77) and excluded (*n* = 68) articles who did not meet eligibility criteria (Fig. 1). Finally, 9 studies were included for qualitative and quantitative synthesis (Fig. 1).

### Statistical analysis

Metafor package of software R [6] version 4.0.2 was used. A proportion meta-analysis technique was performed using the inverse variance method and the Random effects model to estimate the effect. Heterogeneity was quantified by the DerSimonian-Laird Estimator for *τ*^2^ . Outcomes of interest were treated as dichotomous variables, with their respective 95% confidence intervals (95% CI).

## Results

### Study characteristics

We included five case series [7–11], one published RCT [12], and three non-published RCTs [13–15] had the data extracted from the Clinical Trials [16], totaling 149 patients who received cinacalcet. The control group was excluded from the RCTs due to the nature of the work. Patient´s mean age ranged from 35.9 to 204 months. Only two studies included non-dialytic patients [7, 9]. Underlying disease was not reported in the RCTs, and in the case series it was, for the most part, secondary to CAKUT (Congenital Anomalies of the Kidney and Urinary Tract), ranging from 33.33 [7] to 83% [10]. Mean pre-cinacalcet PTH ranged from 932 to 1931 pg/ml (Table 1).

**Table 1:** Clinical and laboratory characteristics of patients included in RCTs and case series

	Alharti A, 2015	Dotis, 2013	Muscheitis, 2008	Silversteim, 2008	Platt, 2010	Warady B, 2019	EUCTR, 2017	NCT 01439867	NCT 02341417
Number	28	4	7	9	6	22	27	18	28
Age (months)	NI	102	204	174	67.2	159.6 (43.2)	153.6 (NI)	35.9 (16.8)	168 (NI)
Gender female (%)	64	50	57.14	33.33	16	54.5	44	33.3	64
CKD stage (%)									
IV	21	0	0	0	0				
V-ND	0	0	14.28	0	0				
V- HD	21	0	42.85	66.66	66	68.2			
V- PD	57	100	42.85	33.33	33	31.8			
Underlying disease (%)						NI	NI	NI	NI
CAKUT	46	50	71.43	33.33	83				
FSGS	21	25	0	11	17				
Other	33	25	28.57	55.55	NI				
Pre PTH (pg/ml)	1931.76 (± 794)	1170 (NI)	932 (NI)	1070 (±171)	970 (NI)	757.1 (± 440)	945.7 (±635)	1299 (634)	1047 (NI)
Pre Calcium (mg/dl)	9.82 (±0.28)	9.55 (NI)	10.18 (NI)	9.4 (±0.2)	9,9 (±0.2)	9.91 (± 0.54)	NI	10.15 (0.76)	9.8 (NI)

Data not informed were described as NI. Age, pre-PTH and pre-Calcium were described as mean and standard deviation. CKD stages were defined as the following: V-ND: stage V CKD non-dialytic; V-HD: stage V CKD in hemodialysis; V-PD: stage V CKD in peritoneal dialysis

### Risk of bias of included studies

**Fig. 2:**
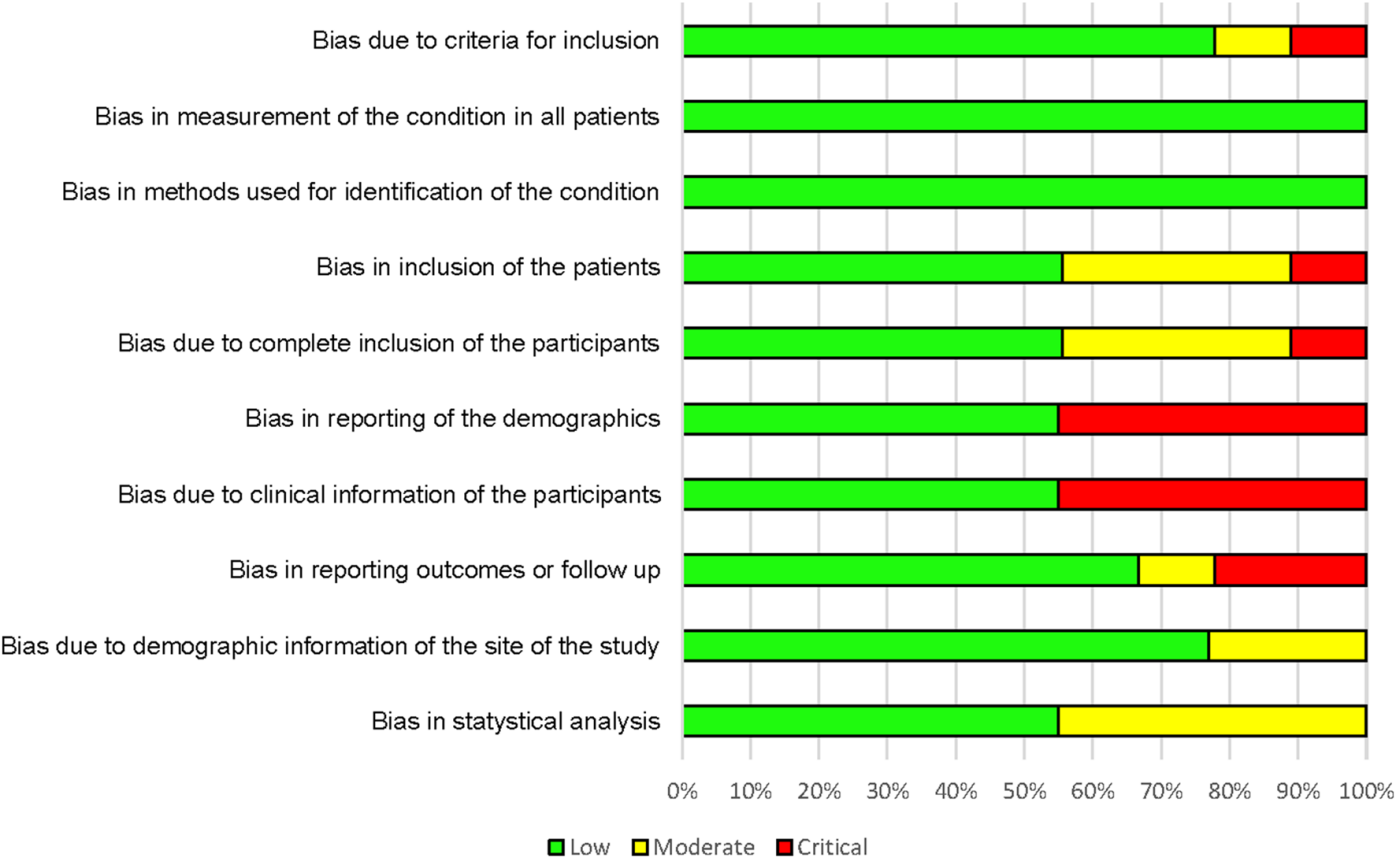
Methodological quality: authors’ assessment of the methodological quality of each item, presented as a percentage of all included studies

Joanna Briggs Institute Collaboration’s tools [17] were used to analyze the risk of bias. Four studies showed severe bias in the presentation of demographic data and clinical information of patients, and two studies showed severe bias in the presentation of outcomes during follow-up, causing bias in the demographic information and clinical characteristics of the patients in 50% of the studies (Fig. 2).

### Results of individual studies

The studies showed important variations in terms of doses (0.2–0.63 mg/kg/day) and duration of therapy (1–24 months) (Table 2). One of the studies did not report the onset of serious or fatal adverse events, 4 reported serious adverse events in 16% of patients to 52.97% and only 2 studies had fatal adverse events as described on Table 2. The serious adverse events were described on Table 3.

**Table 2:** Clinical and laboratory outcomes

	Alharti A, 2015	Dotis, 2013	Muscheitis, 2008	Silversteim, 2008	Platt, 2010	Warady B, 2019	EUCTR, 2017	NCT 01439867	NCT 02341417
Data related to the use of cinacalcet									
Initial dose	0.5	0.25	0.25	NI	0.63	0.18	0.2	0.25	0.2
Maximum dose	NI	0.8	0.25	NI	2.6	0.99	NI	4.2	NI
Mean dose	0.5–1.5	NI	0.25	1.27(±0.3)	NI	1.54 (NI)	0.39	NI	NI
Duration (months)	3–24	5(NI)	1	3 (NI)	15 (NI)	3.6 (NI)	3.7	NI	NI
Adverse events (%)									
Serious adverse events	NI	0	0	0	0	40.9	16	52.97	32
Fatal adverse events	NI	0	0	0	0	4.5	0	0	3
Hypocalcemia	0	0	28.57	0	33	22.7	24	11.8	10.7
Total	0		28.57	33.33	33	81.8	84	82	71

Cinacalcet doses were described as mg/kg/day. Data that had no data were described as NI

**Table 3:** Description of serious adverse events reported in each study

Study	Number	Serious adverse events	Description of the serious adverse events
Alharti 2015	28	NI	6 deaths related to CKD
Dotis 2013	4	0	
Muscheitis 2008	7	0	
Silverstein 2008	9	0	1 patient experienced generalized tonic-clonic seizures after receiving 1 dose of cinacalcet. Serum corrected calcium within the normal range
Platt 2010	6	0	1 patient interrupted treatment ( persistent hypocalcemia)
Warady 2019	22	40.9	Double-blind phase: HAS (9% of patients. n=2) Open-abel phase (n=10): 4 patients (40% reported: esophageal varices, peritonitis, pneumonia,TI, hemoglobin elevation, hypocalcemia (40%, n=4), nausea (30%, n=3), hypertensive encephalopathy and hypertension (20%. n=2) 1 fatal event (double-blind phase): patient with prolonged QT interval at baseline, death occurred in the 23rd week of use, corrected serum calcium 5.3mg/dL
EUCTR 2017	27	16	Hypertension, AVF hemorrhage, diarrhea, ileus, dialysis device displacement, hypervolemia, catheter infection, peritonitis, postoperative wound infection, soft tissue infection (1 patient for each event)
NCT 01439867	18	53	Complication associated with catheter and hypertension (2 patients each); diarrhea, ileus, catheter-related infection, peritoneal dialysis-related complications, dehydration, seizure, catheter malfunction (1 patient each)
NCT 02341417	28	32	Catheter-related infection, displacement of dialysis device (2 patients each): anemia, gout, gastroduodenitis, fatigue, cellulitis, pneumonia, UTI, tachypnea, venous occlusion (1 patient each)

### Summary of results

**Fig. 3:**
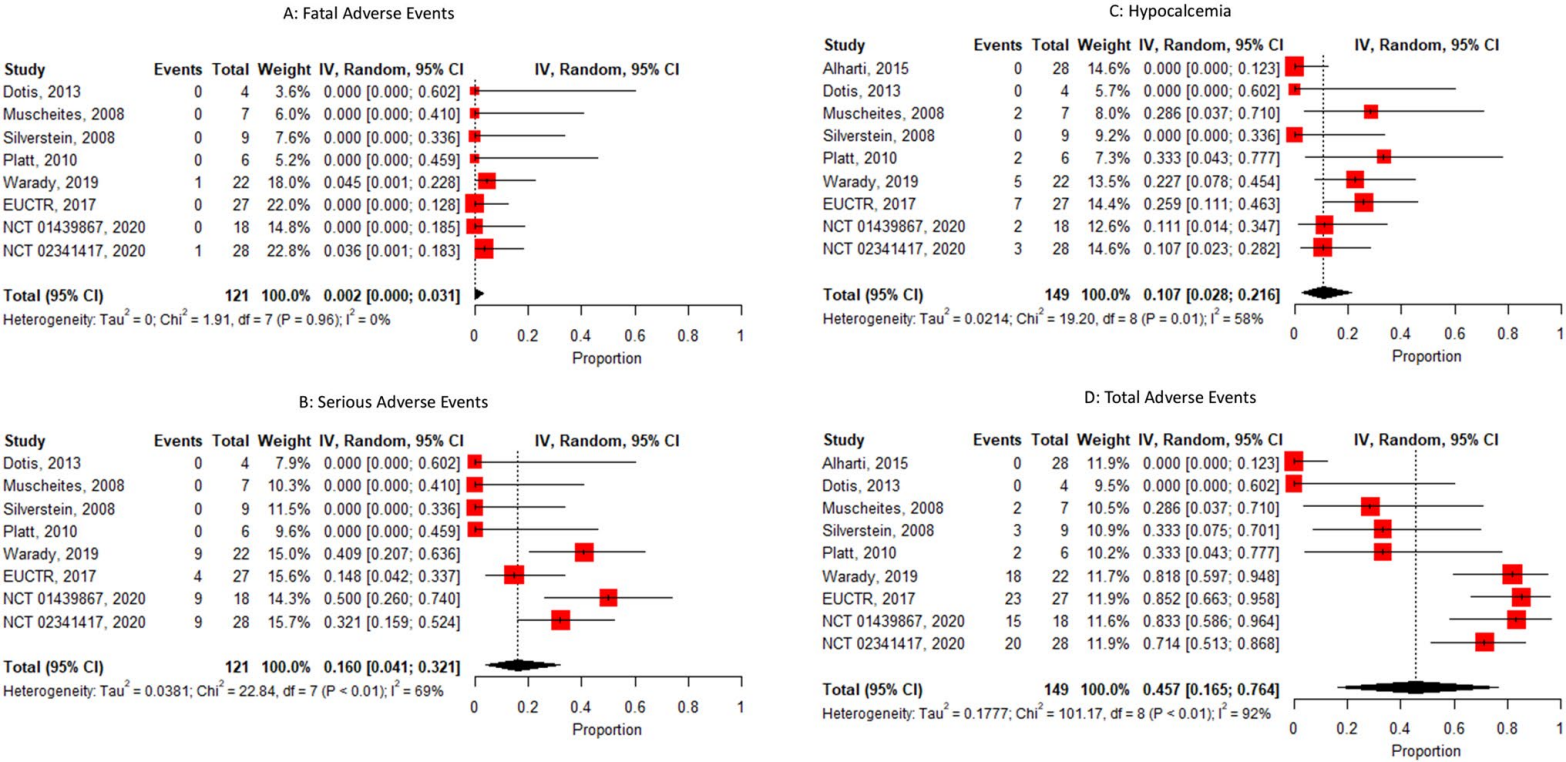
Forest plot (random effect model). **a** Fatal adverse event. **b** Serious adverse event. **c** Hypocalcemia. **d** Total adverse events

We found an incidence of 0.2% fatal adverse event [95% CI 0–3.1%; *I*^2^ = 0%, *p* = 0.96] (Fig. 3a), 16% of serious adverse events [95% CI 4.1–32%; *I*^2^ = 69%, *p* value < 0.01] (Fig. 3b), 10.7% of hypocalcemia [95% CI 2.8–21.6%; *I*^2^ = 58%; *p* value = 0.01] (Fig. 3c), totaling 45.7% of total adverse events [95% CI 16.5–76.4%; *I*^2^ 92%; *p* value < 0.01] (Fig. 3d).

### Additional analysis

**Fig. 4:**
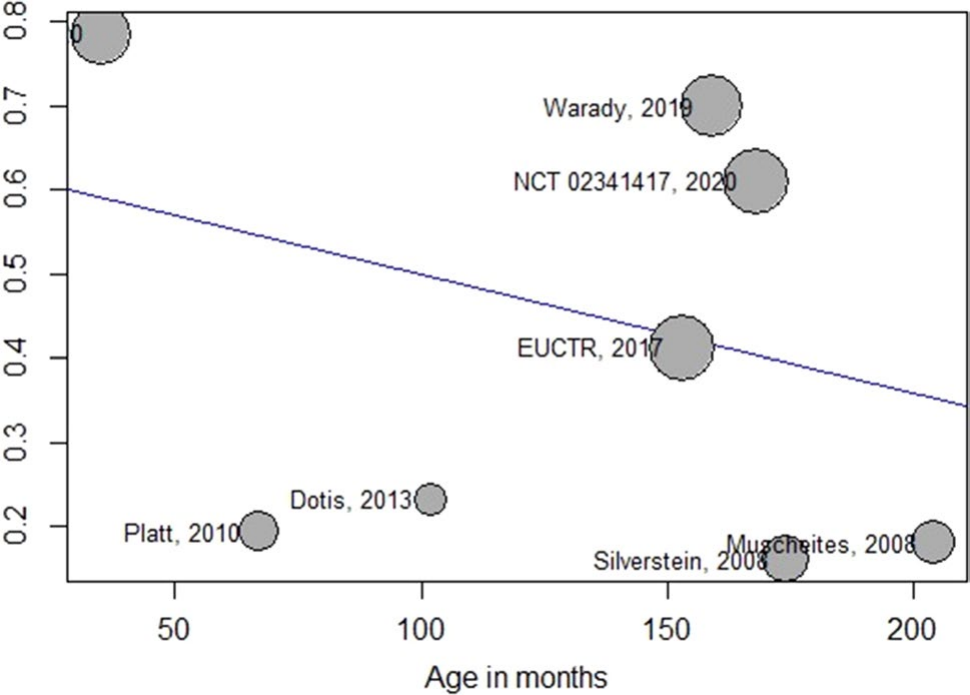
Meta-regression. Age (months) versus serious adverse event (Y-axis). The size of the circle refers to the importance of the study

A meta-regression was performed considering serious adverse event and age in months (Fig. 4). The older the patient, the lower the percentage of serious adverse events (Y-axis) occurred, without reaching significance (*p* = 0.38).

## Discussion

Cinacalcet is a medication widely used to treat BMD in adult patients with CKD. However, safety analyses of cinacalcet in pediatric patients are scarce, limiting its use in this group. In our review, we found an incidence of 0.2% of fatal events reported in two studies and 16% of serious adverse events (*p* < 0.01). Serious adverse events with the highest incidence were hypertension, diarrhea, ileus, and dialysis catheter-related events (Table 3). Three studies reported no serious adverse events but described treatment discontinuation due to persistent hypocalcemia [10], generalized tonic–clonic seizure [11], and six deaths attributed to CKD [7]. The incidence of hypocalcemia and total events were 10.7% (*p* 0.01) and 45.7%, respectively.

A systematic review conducted by Ballinger et al. [18] showed an increased risk of hypocalcemia in adults on dialysis who received cinacalcet (12 studies, 6415 participants, RR 6.98, 95% CI 5.10–9.53; *I*^2^ = 0%).

In the EVOLVE trial [19] hypocalcemia was found in 12% and 1.7% in the cinacalcet and placebo groups, respectively. The percentage of treatment-related serious adverse events was similar between the groups (3.6% and 2.3%, respectively).

Four RCTs [20–23] reported no serious adverse events and an average percentage of reduction in calcium values of 4% [23], 6.8% [20] and 4.7% [22]. Most adverse events were considered mild to moderate in these studies and transient episodes of hypocalcemia in patients who received cincacalcet were reported in one study [21].

The incidence of hypocalcemia found in the present study was similar to that reported in the adult population [19–23]; however, serious adverse events were five times higher. Additionally, two deaths were reported in the pediatric population, but it was not possible to rule out cinacalcet as a causal factor [12, 15, 24, 25] Two studies that reported high rates of adverse events [14, 15] were not published but had data retrieved from the Clinical Trials platform [16].

The main side effects of cinacalcet are the gastrointestinal intolerance and the potential incidence of symptomatic hypocalcemia, so caution should be exercised in patients with risk factors to present a interval QT prolongation or patients with epilepsy. A certain degree of asymptomatic hypocalcemia induced by calcimimetics is considered tolerable and could even be beneficial. In addition, with a relatively low calcium, FGF23 decreases, as long as phosphate is controlled [26, 27].

Warady et al. [24] performed a recent comprehensive review. Cinacalcet pharmacokinetics data are similar between pediatric and adult subjects with CKD and secondary HPT receiving dialysis and between pediatric age groups (28 days to < 6 years and 6 years to < 18 years). The most common adverse events (occurring in > 10% of subjects) were hypocalcemia (22.8%), vomiting (16.5%), nausea (15.2%), systemic hypertension (11.4%), pyrexia (10.1%), and muscle spasms (10.1%).

Calcimimetics may be considered with extreme caution in infants who have persistent and severe hyperparathyroidism in the presence of high or high-normal calcium levels, despite optimized conventional management, including active vitamin D, as an alternative to parathyroidectomy in individual cases after informed consent of the family, provided a close follow-up of ionized Ca and Ca levels and the subsequent risk of hypocalcemia [24]. A closer monitorization may be necessary in patients under treatment with calcimimetics, especially during the period of dose adjustment [26].

We found high rates of serious adverse events, but the main serious events reported were hypertension, diarrhea, and dialysis catheter-related events. In addition, the meta-regression (Fig. 4) indicates that the younger the age, the higher the incidence of adverse events. Despite not reaching statistical significance, possibly due to the reduced number of cases, the incidence of serious adverse events can reach 80% at 50 months (Fig. 4).

This study is limited by the number of participants and studies nature (case series). However, this is the first systematic review with a proportional meta-analysis of case series on the safety of cinacalcet use in children and adolescents with hyperparathyroidism secondary to CKD. Additionally, we expanded the search to gray literature sources to include unpublished works that had data retrieved.

## Conclusion

If used in the pediatric population, cinacalcet should have careful monitoring of serum calcium levels and attention to possible adverse events, especially in children younger than 50 months.  

## Data Availability

The data used to support the results and conclusion of this manuscript were presented by the authors.
